# Establishment and clinical performance evaluation of the thyroglobulin colloidal gold immunochromatographic assay

**DOI:** 10.3389/fonc.2026.1786894

**Published:** 2026-08-17

**Authors:** Qi Yang, Jifeng Li, Jinzi Hui, Jia Dai, Hongbian Gao, Yuan An

**Affiliations:** 1 Shaanxi Provincial Cancer Hospital Affiliated to Xi’an Jiaotong University, Head and Neck Oncology Department, Shaanxi, China; 2 Shaanxi Provincial Cancer Hospital, PR Head and Neck Oncology Department, Shaanxi, China; 3 Shaanxi Provincial Cancer Hospital, Department of Nuclear Medicine, Shaanxi, China; 4 Shaanxi Provincial Cancer Hospital Affiliated to Xi’an Jiaotong University, Department of Nuclear Medicine, Shaanxi, China; 5 Clinical Laboratory of Shaanxi Provincial Cancer Hospital, Shaanxi, China; 6 Shaanxi Provincial Cancer Hospital Affiliated to Xi’an Jiaotong University, Clinical Laboratory, Shaanxi, China; 7 Shaanxi Provincial Cancer Hospital, Department of Pathology Medicine, Shaanxi, China; 8 Shaanxi Provincial Cancer Hospital Affiliated to Xi’an Jiaotong University, Department of Pathology Medicine, Shaanxi, China

**Keywords:** clinical performance evaluation, colloidal gold immunochromatographic assay, differentiated thyroid cancer, lymph node metastasis, thyroglobulin

## Abstract

**Background:**

Lymph node metastasis increases recurrence risk in differentiated thyroid carcinoma (DTC). To address limitations of conventional detection, this study developed a novel quantitative diagnostic protocol to enhance preoperative assessment accuracy and guide optimal surgical management.

**Methods:**

A total of 185 serum samples and 120 lymph node samples from patients scheduled for thyroid lateral neck lymph node resection or biopsy at the Head and Neck Surgery of Shaanxi Provincial Cancer Hospital between July 2023 and December 2023 were detected through the colloidal gold immunochromatographic assay (CGICA) and traditional Roche electrochemiluminescence immunoassay (ECLIA) methods, respectively. The paraffin pathology results served as the gold standard.

**Results:**

The CGICA method demonstrated comparable performance to the Roche ECLIA method in serum sample detection, as evidenced by excellent correlation in regression analysis (r=0.996) and Bland-Altman agreement analysis. Notably, when applied to lymph node specimens, the CGICA method with a 22.05 μg/L cutoff value exhibited greater sensitivity than the Roche method with a 233.25 μg/L cutoff value (90% *vs* 86.25%). Importantly, the CGICA method achieved a diagnostic accuracy comparable to that of intraoperative frozen section histopathology while offering technical advantages in rapid thyroglobulin quantification via fine-needle aspiration biopsies.

**Conclusions:**

The current study demonstrates the potential of the CGICA method for thyroglobulin detection, which contributes to the rapid detection of cervical lymph node metastases in thyroid cancer patients (clinical trial registry number: ChiCTR2200063561).

## 1 Background

The incidence of thyroid cancer has increased significantly over the past several decades and has attracted widespread attention (1). Extensive evidence has established that differentiated thyroid cancer (DTC) accounts for approximately 90% of all thyroid cancer cases, among which​ papillary thyroid carcinoma (PTC) is the predominant subtype (2). Although PTC is generally associated with a more favorable prognosis (3), approximately 40% - 60% of patients experienced early cervical lymph node metastases. Regrettably, lymph node metastasis confers a significantly elevated risk of recurrence and thyroid cancer-related mortality in individuals (4). Lymph node status, especially lateral cervical lymph node involvement, serves as a core determinant for clinical staging and the formulation of surgical strategies.

Currently, the available modalities for identifying lymph node metastasis encompass the thyroglobulin measurement with fine-needle aspiration (FNA-Tg) (5), intraoperative frozen section analysis (6) and postoperative paraffin pathology (7). However, frozen section diagnosis with relatively high accuracy relies on sophisticated equipment and specialist pathologists, thus posing obstacles to clinical application at primary hospitals (8). In addition, conventional assays for FNA-Tg detection primarily comprise radioimmunoassay (RIA), immunoradiometric assay (IRMA), enzyme linked immunosorbent assay (ELISA) and electrochemiluminescence immunoassay (ECLIA) (9), among which ECLIA represents the predominant modality for thyroglobulin (Tg) testing owing to its outstanding repeatability, sensitivity and stability (10). However, the ECLIA modality requires bulky instrumentation and prolonged assay workflows, hindering timely intraoperative diagnosis of suspicious lymph nodes (11). Accordingly, developing a rapid and accurate quantitative assay for intraoperative lymph node metastasis detection represents an urgent clinical requirement to optimize the comprehensive therapeutic performance of DTC surgery.

The growing emphasis on biomarker-guided clinical decision-making provides a compelling rationale for developing rapid, point-of-care biomarker assays. For instance, a recent study has demonstrated that longitudinal monitoring of biochemical markers following bariatric metabolic surgery is essential for optimizing perioperative management and improving patient outcomes (12). For patients diagnosed with DTC, Tg acts as a pivotal biomarker to screen for cervical lymph node metastasis. Immunochromatographic test strips (ICS) drastically reduce turnaround time for detection, rendering them ideal for intraoperative point-of-care testing (13). The CGICA assay operates based on the direct correlation between the color intensity of the test line on the nitrocellulose membrane and target analyte concentrations (14), among which the T/C value (the ratio of test line signal intensity to control line signal intensity) serves as a widely adopted quantitative indicator. In addition, the CGICA assay boasts additional merits such as low cost and straightforward preparation (11). Accordingly, the CGICA method for FNA-Tg detection holds promising prospects for widespread clinical translation.

This study aimed to establish a novel CGICA assay for rapid Tg detection, which could offer the advantages of quantification, simplicity and time-savings. The accuracy and clinical performance of the CGICA method were assessed to provide support for its clinical application. Collectively, the newly developed CGICA method enables intraoperative diagnosis of lymph node metastasis within mere minutes.

## 2 Methods

### 2.1 Collection and processing of clinical samples

**Figure 1:**
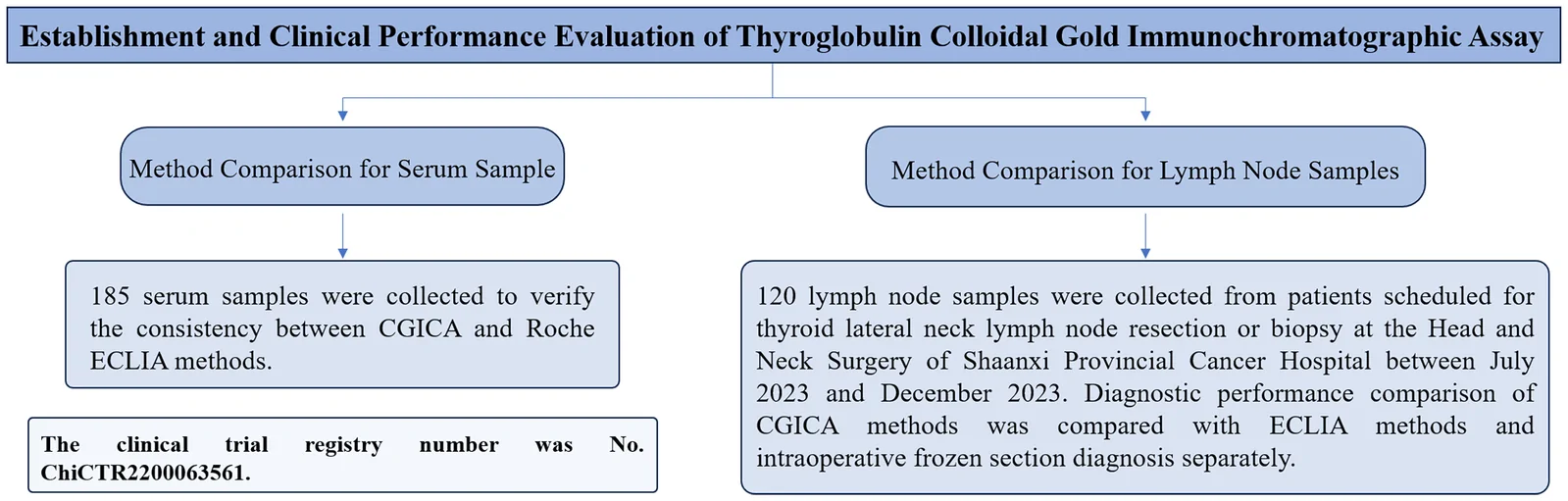
Clinical research design and procedures.

This study was approved by the ethics committee of Shaanxi Provincial Cancer Hospital (5101128) and registered in the Chinese Clinical Trial Registry (No. ChiCTR2200063561). All procedures were conducted in accordance with the principles of the Declaration of Helsinki. All participants in the study received the written informed consent forms. The study design and procedures were shown in **Figure 1**.

For the serum samples, 185 samples were obtained from the remaining serum samples of patients who completed necessary testing at Shaanxi Provincial Cancer Hospital. The inclusion criteria were as follows: 1. The remaining samples were collected after daily testing in the laboratory of our hospital. 2. The background information of the sample was clear and traceable without the limits of the patient’s age and gender. 3. No less than 300 μl of sample. 4. The serum and plasma samples were clear and light yellow with no visible turbidity or insoluble substances or obvious hemolysis except for the interfering samples. The exclusion criteria were as follows: 1. The background information of the sample was not clear or traceable. 2. The same type of sample from same patient. 3. Samples with severe hemolysis, lipemia or icterus. 4. The samples that the investigator considered did not meet the test requirements. 5. The samples that did not meet the sample collection, processing and preservation methods specified in the scheme. The sample exclusion criteria were as follows: 1. The samples that could not complete the test because of instrument or human factors (sample contamination during operation, etc.). 2. The samples whose test values were outside the linear range of the experimental reagents or the comparison reagents. All samples were detected via the CGICA and Roche ECLIA methods.

For the lymph node samples, a total of 120 patients scheduled for thyroid lateral neck lymph node resection or biopsy at the Head and Neck Surgery of Shaanxi Provincial Cancer Hospital between July 2023 and December 2023 were selected. The average age of the patients was 38.8 ± 10.8 years, 70 PTC patients were females (58.33%) and 50 PTC patients were males (41.67%). All patients underwent initial surgery and had complete clinical data. After eliminating other potential causes of cervical lymph node enlargement, 69 patients underwent cervical lymph node dissection during surgery and 51 patients underwent excisional biopsy of suspected enlarged lymph nodes in the cervical region. Lymph nodes were regarded as suspicious upon satisfying no fewer than two of the following sonographic markers: 1) long-to-short axis ratio > 1, 2) size criteria (long diameter > 8 mm for lateral neck nodes and > 5 mm for central compartment nodes), 3) specific ultrasound features (internal hypoechogenicity, microcalcifications, cystic degeneration), 4) hilum effacement or eccentric hilum. All the samples were subjected to paraffin pathological examination postsurgery. For lymph node sampling, a 5 mL hollow-core needle with a 23 G tip was used to perform multipoint puncture on the dissected target lymph node under direct vision, followed by repeated aspiration with 1 mL of physiological saline to obtain the sample eluent. Each lymph node punctured was labeled separately and subjected to the rapid frozen section pathology and routine pathological examination.

### 2.2 Detection of Tg

Sample serum and eluent were added to the sample well of the Tg detection card for CGICA detection (Suzhou Dongni Biotechnology Co., Ltd., China), which was also further labeled for the Roche ECLIA assay (Roche Diagnostics GmbH, Germany). The tested samples in the Tg detection card flowed to the absorption pad due to capillary action. Tg in the sample initially bound to the precoated CG antibody conjugates on the conjugate pad. Followed by the capture by the Tg antibody located in the test zone to form the red ribbon. The intensity of the band color was directly proportional to the Tg levels of the sample within a defined range. The control line was precoated with anti-streptavidin antibodies and consistently turned wine red. For the image with a colored signal on the immunochromatographic strip after waiting for 1 min, both the test and control lines were red, the sample was recorded as positive. The red control line with no change in the test line was recorded as negative. Quantitative analysis was performed on the strip via an Immunity Ration Detector (Ma’anshan Ruiheng Technology Co., Ltd., China) after 3 min. The Tg concentration measured by the instrument ranged from 0.05 to 500 ng/mL.

### 2.3 Outlier analysis

Each pair of test results was calculated as follows (Equations 1–4), in which y_i_ was the detection value of the experimental reagents and x_i_ was the detection value of the comparison reagents. If D_i_ > 4E and D_i_’ > 4E’, the corresponding value was considered as a discrete outlier.

(1) $$D_{i}=|y_{i}-x_{i}|$$

(2) $${\text{D }}_{i}^{'}=|y_{i}-x_{i}|/x_{i}$$

(3) $$\text{E}=\frac{1}{n}\sum_{\text{i}=1}^{\text{n}}D_{i}$$

(4) $$\text{E'}=\frac{1}{n}\sum_{\text{i}=1}^{\text{n}}D_{i}^{"}$$

### 2.4 Statistical analysis

All the statistical analyses were carried out via IBM SPSS 24.0 software and GraphPad Prism 9 software. The measurement data were presented as the means ± SD. The chi-square test was used for comparisons between groups. A Z-test was used to compare the area under the curve (AUC) of the receiver operating characteristic (ROC) between the two methods. The optimal cut-off points for FNA-Tg for diagnosing lymph node metastasis were calculated via the Youden index. The consistency was evaluated via the Bland-Altman method. *P* values were derived from two-tailed tests. The results for which *p* was < 0.05 were considered significant.

## 3 Results

### 3.1 Comparison between the CGICA and ECLIA methods

#### 3.1.1 Outlier analysis

As shown in **Table 1**, the maximum value of D_i_ (6.550) was less than 4E (7.612), indicating no outliers.

**Table 1:** Outlier analysis.

Metric	Minimum value	Maximum value	E	SD^a^
D_i_	0.070	6.550	1.903	1.180
D_i_’	0.000	0.890	0.128	0.165

a Standard deviation.

#### 3.1.2 Regression analysis

**Figure 2:**
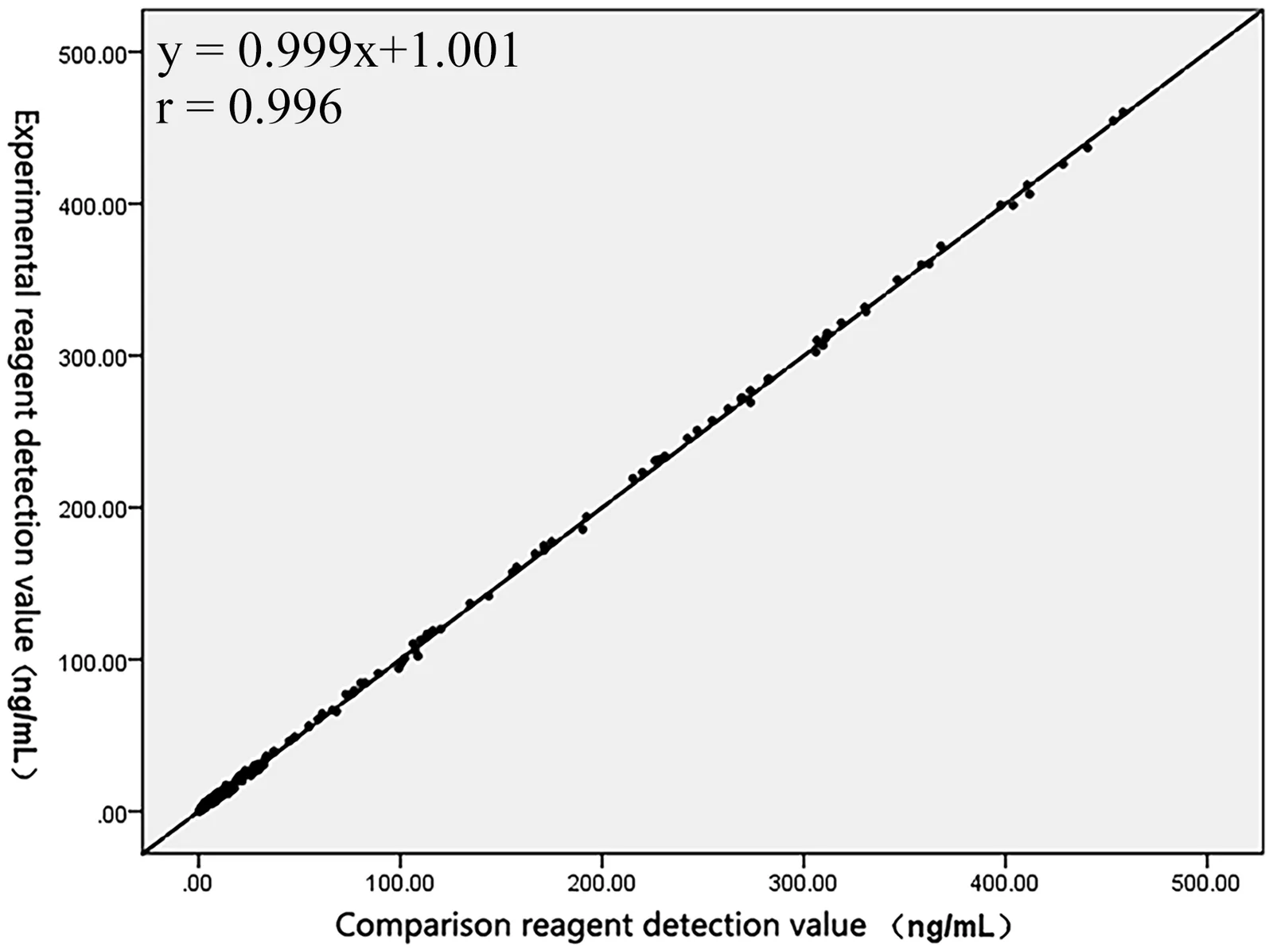
Scatter plot of the detection values. The x-axis represented the detection values of the ECLIA method and the y-axis represented the detection values of the CGICA method.

In the scatter plot (**Figure 2**), the x−axis represented the detection value of the ECLIA method and the y−axis represented the detection value of the CGICA method. The following linear regression equation was obtained: y = 0.999x+1.001. Spearman correlation analysis was used to calculate the rank correlation coefficient (r = 0.996). The results suggested great agreement between the two measurement methods.

#### 3.1.3 Consistency analysis

**Figure 3:**
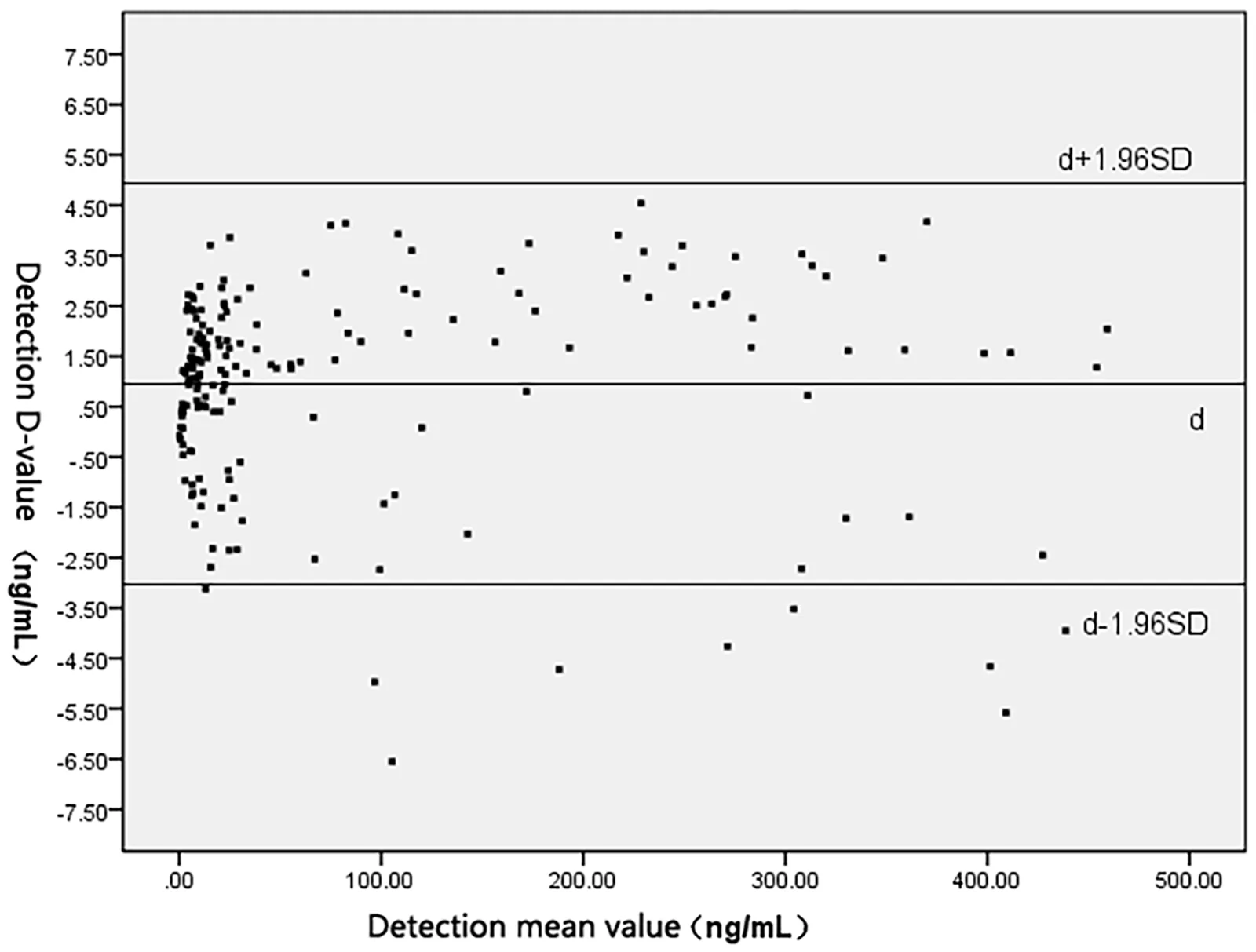
Scatter diagram of the consistency analysis. The x-axis represented the difference (D) for each pair of measurements and the y-axis represented the average (mean) for each pair of measurements. The 95% limits of agreement were equivalent to D ± 1.96 standard deviations (SDs).

The consistency between the CGICA and ECLIA methods was verified via Bland-Altman analysis. As shown in **Figure 3**, the difference (D, x-axis) and the average (mean, y-axis) were calculated for each pair of measurements. The 95% limit of agreement ranged from -3.033 to 4.931. 9 points fell outside the limits of agreement, and the other 176 points fell within, again confirming acceptable consistency between the two methods.

### 3.2 Assessment of diagnostic performance

#### 3.2.1 Results of lymph node diagnosis

A total of 120 lymph node samples were collected. 11 patients were diagnosed as negative via intraoperative frozen section, but the actual result was positive according to postoperative paraffin pathology. Furthermore, 77 (64.17%) lymph nodes were diagnosed as metastatic and 43 (35.83%) lymph nodes were benign via paraffin pathology.

#### 3.2.2 Diagnostic performance comparison between the CGICA and ECLIA methods

**Figure 4:**
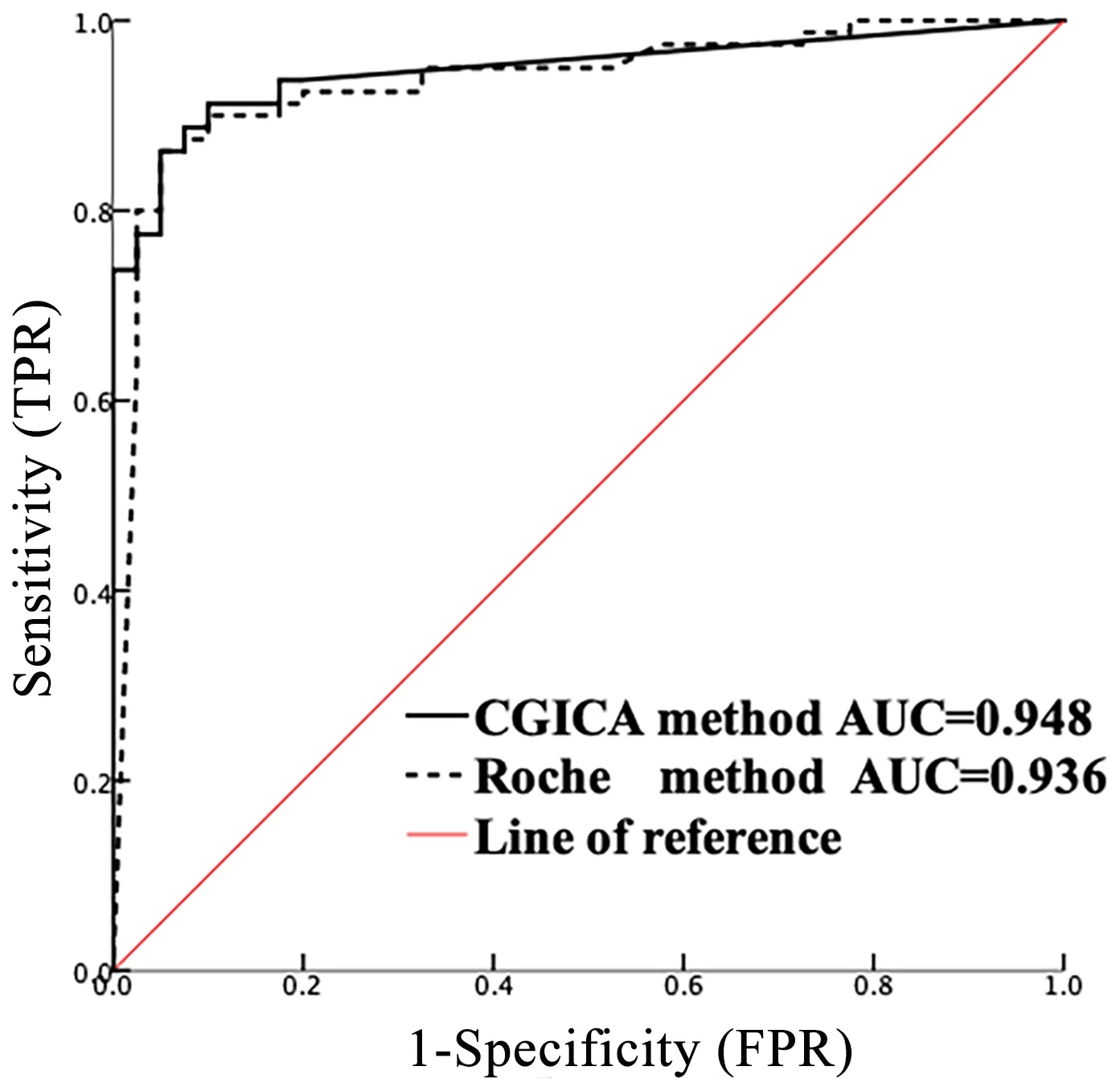
ROC curves of the two methods for detecting FNA-Tg in cervical lymph nodes. The x-axis represented the values of false positive rate (FPR) and the y-axis represented the values of true positive rate (TPR).

Paraffin pathology results served as the gold standard. The ROC curves were presented in **Figure 4**. The AUC of the CGICA method was 0.948 with a sensitivity of 90% and a specificity of 85%. The AUC of the Roche ECLIA method was 0.936 with a sensitivity of 86.25% and a specificity of 95%. There was no statistically significant difference in the AUC between the two methods according to the Z-test (Z = 0.0384, *p* = 0.512). Further analysis revealed that the optimal cutoff value was 22.05 μg/L for the CGICA method and 233.25 μg/L for the Roche method, respectively. The diagnostic performance of the two methods was analyzed based on the corresponding cut-off values in **Table 2**. The McNemar test indicated that there was no statistically significant difference in diagnostic outcomes between the CGICA and Roche methods (*p* > 0.05). However, the sensitivity and specificity of both methods were statistically significant (χ^2^ = 82.286, *p* < 0.05) according to the extended paired χ² test. The CGICA method exhibited greater sensitivity than the Roche method (χ^2^ = 48.12, *p* = 0.000). The specificity showed no statistically significant variation (χ^2^ = 0.526, *p* = 0.468).

**Table 2:** Comparison of diagnostic performance between CGICA and Roche ECLIA methods.

Methods	CGICA method	Roche ECILA method
Number of samples (case)	120	120
Diagnostic cutoff (ng/mL)	22.05	233.25
Positive (case)	72	69
False positive (case)	6	2
Negative (case)	34	38
False negative (case)	8	11
Sensitivity (%)	90	86.25
Specificity (%)	85	95
Accuracy rate (%)	88.33	89.17
Positive predictive value (%)	92.31	97.18
Negative predictive value (%)	80.95	77.55

#### 3.2.3 Diagnostic performance comparison between the CGICA method and intraoperative frozen section diagnosis

Similarly, paraffin pathology results served as the gold standard. The diagnostic performance of the CGICA method for FNA-Tg detection and intraoperative frozen section diagnosis were separately calculated separately (**Table 3**). The sensitivity and specificity of the CGICA method were 91.25% and 85%, respectively. The sensitivity and specificity of intraoperative frozen section diagnosis were 86.25% and 100%, respectively. There was no statistically significant difference in the diagnostic performance of the two methods according to the McNemar test (*p* = 0.096). Furthermore, the sensitivity and specificity also showed no statistically significant variation according to the extended paired χ² test (χ^2^ = 1.421, *p* = 0.233).

**Table 3:** Comparison of diagnostic performance between CGICA methods and intraoperative frozen section diagnosis.

Methods	CGICA Method (Diagnostic cutoff = 22.05 ng/mL)	Frozen section diagnosis
Number of samples (case)	120	120
Positive (case)	73	69
False positive (case)	6	0
Negative (case)	34	40
False negative (case)	7	11
Sensitivity (%)	91.25	86.25
Specificity (%)	85	100
Accuracy rate (%)	89.17	66.7
Positive predictive value (%)	92.41	100
Negative predictive value (%)	82.93	78.43

## 4 Discussion

FNA-Tg has shown high diagnostic accuracy and achieved favorable sensitivity and specificity for identifying lymph node metastases in patients with PTC (15). The 2025 American Thyroid Association guidelines further endorses​ FNA-Tg as a supplementary tool​ to FNA cytology for evaluating sonographically suspicious lymph nodes (2). However, as the most widely applied technique, the Roche assay requires costly equipment, experienced operators and several hours of turnaround time (11), limiting its availability in primary hospitals. Consequently, it is necessary to develop a novel affordable, convenient, rapid and accurate method for FNA-Tg measurement.

Preliminary studies have suggested the clinical potential of CGICA as a rapid assay for intraoperative FNA-Tg detection (11, 16). To further investigate the clinical applicability of the CGICA method, regression analysis and consistency analysis were carried out between the CGICA and ECLIA methods. The distribution of 185 collected serum samples ranged from 0.05–500 ng/mL (**Supplementary Materials**). As expected, favorable consistency was observed between Tg measurements obtained using the ECLIA and CGICA assays. Considering the inherent limitations of the ECLIA method, CGICA may serve as an excellent alternative method for the rapid detection of serum Tg.

In fact, the CGICA method had also shown great potential for application in FNA−Tg determination. Compared with the Roche method, the developed CGICA method exhibited greater sensitivity (90%) for FNA-Tg detection, which may be attributed to the increased reaction surface area. Specifically, expanding the reactive surface area effectively strengthens interactions with target analytes (17), thereby improving sensitivity and potentially compensating for false−positive results caused by cross−reactions in the Roche method. Currently, intraoperative frozen section serves as a valuable assay in surgical decision-making for DTC, enabling identification of malignant foci and lymph node metastases (18). Intraoperative rapid frozen pathology detection achieved a sensitivity of 86.25% and a specificity of 100% in the current research, which is consistent with the previously reported results (19–22). Interestingly, the sensitivity of the CGICA method was 91.25%, which was clearly superior to that of intraoperative frozen section diagnosis. However, the CGICA assay yielded a specificity of 85%, suggesting that the current platform retains scope for further optimization.

There was no significant difference in sensitivity or specificity between the CGICA method and frozen section diagnosis based on the results of the extended paired χ² test and the McNemar test. However, the present study identified 9 patients who tested positive with the CGICA assay yet negative on frozen pathological examination. The reason for this phenomenon was that the cancer lesion was smaller than 2 mm. In fact, the eluent prepared by multipoint puncture of the lymph node reflects the overall situation of the entire lymph node rather than just the local area, thereby avoiding potential misjudgments caused by objective factors such as slice quality. Additionally, 6 cases were diagnosed as positive by the CGICA method, but negative by frozen pathology and postoperative paraffin pathology, possibly due to specimen contamination caused by glandular removal prior to lymph node dissection. Thus, it is extremely advisable for the CGICA method to minimize contact between the lymph nodes and thyroid glands, including the new instruments should be used whenever possible during lateral neck dissection. Furthermore, the CGICA method required less time than frozen section diagnosis. The duration from preparation of the eluent to detection did not exceed 10 min for the CGICA method.

Additionally, there were several limitations in the current study. 1) more diverse case types should have been included, rather than only patients diagnosed with PTC and suspected lateral neck lymph node metastasis via fine−needle aspiration. 2) discrepancies may exist between surgically resected lymph nodes and those identified on ultrasound, partly attributable to variations in surgeons’ operative experience. 3) further enlargement of clinical sample size is required in subsequent studies.

## 5 Conclusions

A novel CGICA approach used for the semi-quantitative detection of the thyroid cancer biomarker Tg in lymph nodes was proven effective in the current study. Compared with the Roche method, the CGICA method for FNA-Tg detection exhibited greater sensitivity. Furthermore, there was no significant difference in diagnostic performance between the CGICA method and the Roche method or frozen section diagnosis. The advantages of the CGICA method, including its high sensitivity, wide detection range, rapid detection time and convenient operation, enable surgeons to efficiently assess the necessity and scope of lymph node dissection in thyroid cancer surgery.

## Data Availability

The original contributions presented in the study are included in the article/**Supplementary Material**. Further inquiries can be directed to the corresponding author.
