# Supplementary material for: Establishment and clinical performance evaluation of the thyroglobulin colloidal gold immunochromatographic assay
Source: Front Oncol. 2026 Aug 17;16:1786894. doi: 10.3389/fonc.2026.1786894 (PMC13526582; doi:10.3389/fonc.2026.1786894)
Supplement: Supplementary file 1 [file Table1.docx]

**Supplementary materials**

The distribution of 185 serum samples was shown in **Table S1** and the concentrations of all the samples fell within the linear range of the CGICA method.

Table S1 Frequency distribution of samples

| **Concentration (ng/mL)** | 0.05 | 0.05~3.5 | 3.5～77 | 77～500 | >500 |
| --- | --- | --- | --- | --- | --- |
| **Number of samples (case)** | 0 | 22 | 102 | 61 | 0 |
| **Frequency (%)** | 0 | 11.9% | 55.1% | 33.0% | 0 |
